# Concomitant Use of Botulinum Toxin and Super Long Nd:YAG Laser

**DOI:** 10.1111/jocd.70042

**Published:** 2025-03-07

**Authors:** Jernej Kukovič, Anže Zorman

**Affiliations:** ^1^ Medilase d.o.o Ljubljana Slovenia

**Keywords:** botulinum toxin, Nd:YAG laser, PIANO

## Abstract

**Background:**

Botulinum toxin is a widely utilized minimally invasive therapeutic option with minimal downtime and a low risk of serious complications. The long pulse Nd:YAG laser treatment (PIANO) has been utilized in recent years for skin rejuvenation and tightening, body shaping, and transdermal lipolysis through bulk heating of the tissue. The heat‐labile bond between the heavy and light chains of botulinum toxin could be affected by laser bulk heating.

**Aims:**

This case series aims to clinically evaluate the efficacy of botulinum toxin when used in conjunction with PIANO 1064 nm bulk heating.

**Patients/Methods:**

This case series included two patients who underwent standard hyperhidrosis treatment with botulinum toxin and then single‐sided concomitant long‐pulsed Nd:YAG laser treatment.

**Results:**

The iodine–starch test revealed no differences between the two areas at follow‐up visits.

**Conclusion:**

This small study corroborates the findings of other research indicating that long‐pulsed 1064 nm Nd:YAG laser treatment does not affect the efficacy of botulinum toxin.

## 1 Introduction

Botulinum toxin is a widely utilized therapeutic option, not only for treating facial rhytids [1] but also for addressing hyperhidrosis [2], facial palsy [3], bruxism [4], migraines [5], and other conditions. This procedure is attractive to both doctors and patients due to its minimally invasive nature, minimal downtime, and low risk of serious complications [6]. Botulinum toxin is a neurotoxic protein complex that inhibits acetylcholine release at the presynaptic neuromuscular junction. The active molecule comprises heavy and light chains linked by heat‐labile disulfide bonds and noncovalent forces. Both chains are essential for the toxin to enter the cell [7, 8].

Laser procedures are another group of widely used therapeutic options in esthetic medicine [9]. Nonablative laser treatments, in particular, are increasingly favored for skin rejuvenation due to their lack of posttreatment downtime and minimal aftercare requirements compared to ablative laser procedures [10]. Various nonablative wavelengths are employed to improve the appearance of wrinkles, skin texture, acne scars, and pigmentary and vascular changes [11]. The exact mechanisms of nonablative dermal remodeling are still under investigation; however, existing research indicates that subthreshold laser‐induced injury to the dermis and/or dermal vasculature triggers a wound repair response, fibroblast recruitment and stimulation, and collagen reformation [12]. Since thermal energy is the source of this injury, most non‐ablative devices target different chromophores in the dermis, which contribute to the tissue heating through selective photothermolysis and the resulting regenerative response [11].

Botulinum toxin is known to be inactivated at 85°C for 5 min. On the other hand, the effect of lower temperatures produced during various nonablative laser therapies on the heat‐labile bond between the heavy and light chains of botulinum toxin remains unclear [8, 13]. Some studies have shown no change or even an increase in botulinum toxin efficacy when combined with both ablative [14, 15] and nonablative lasers [16, 17, 18, 19]. The 1064 nm Nd:YAG laser has also been studied in this context, though not with the super long, bulk heating PIANO modality [20, 21, 22].

The PIANO laser treatment (Fotona, Slovenia) has been utilized in recent years for skin rejuvenation and tightening, body shaping, and transdermal lipolysis [20, 21, 23, 24, 25]. Standard rejuvenation protocols, including PIANO rejuvenation and Fotona 4D, typically achieve dermal temperatures of approximately 45°–47° [22]. According to Semchyshyn [16], these temperatures are not high enough to inactivate botulinum toxin.

As the administration of multiple treatments within a single session has become a standard practice in esthetic medicine, this case series aims to clinically evaluate the efficacy of botulinum toxin when used in conjunction with PIANO 1064 nm treatment.

## 2 Cases

The study included a 35‐year‐old Caucasian male and a 33‐year‐old Caucasian female with the complaint of axillary hyperhidrosis that has not been addressed in any surgical way previously. Written consent was received from both participants.

A standard starch iodine test was performed before botulinum toxin treatment for hyperhidrosis [26]. The area of excessive sweating was marked. The abobotulinumtoxinA (Dysport, IPSEN Biopharmaceuticals, France) was diluted to a final concentration of 100 IE per 5 mL syringe.

**FIGURE 1:**
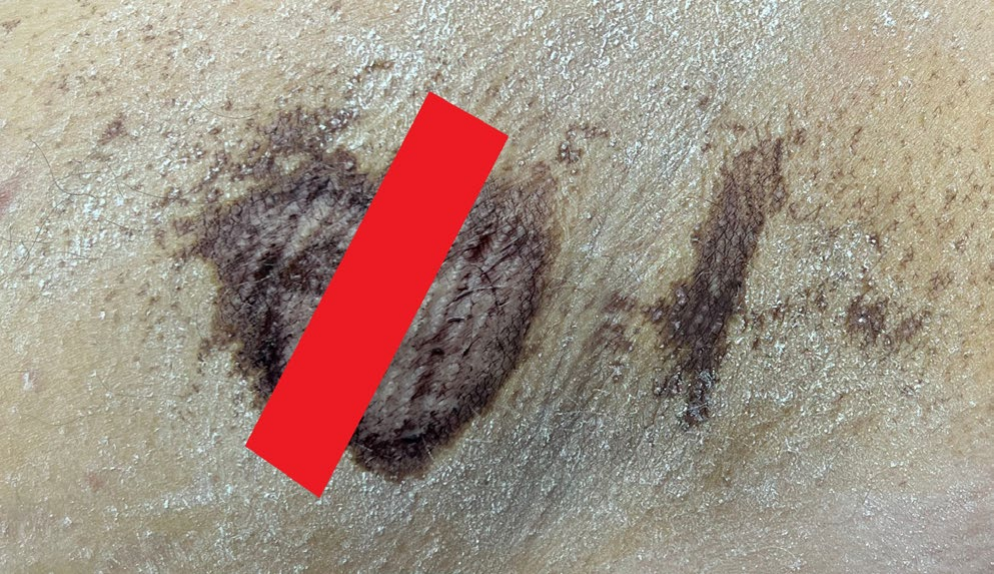
Male patient: Left axilla after iodine–starch test: A schematic of PIANO treatment (marked in red).

**FIGURE 2:**
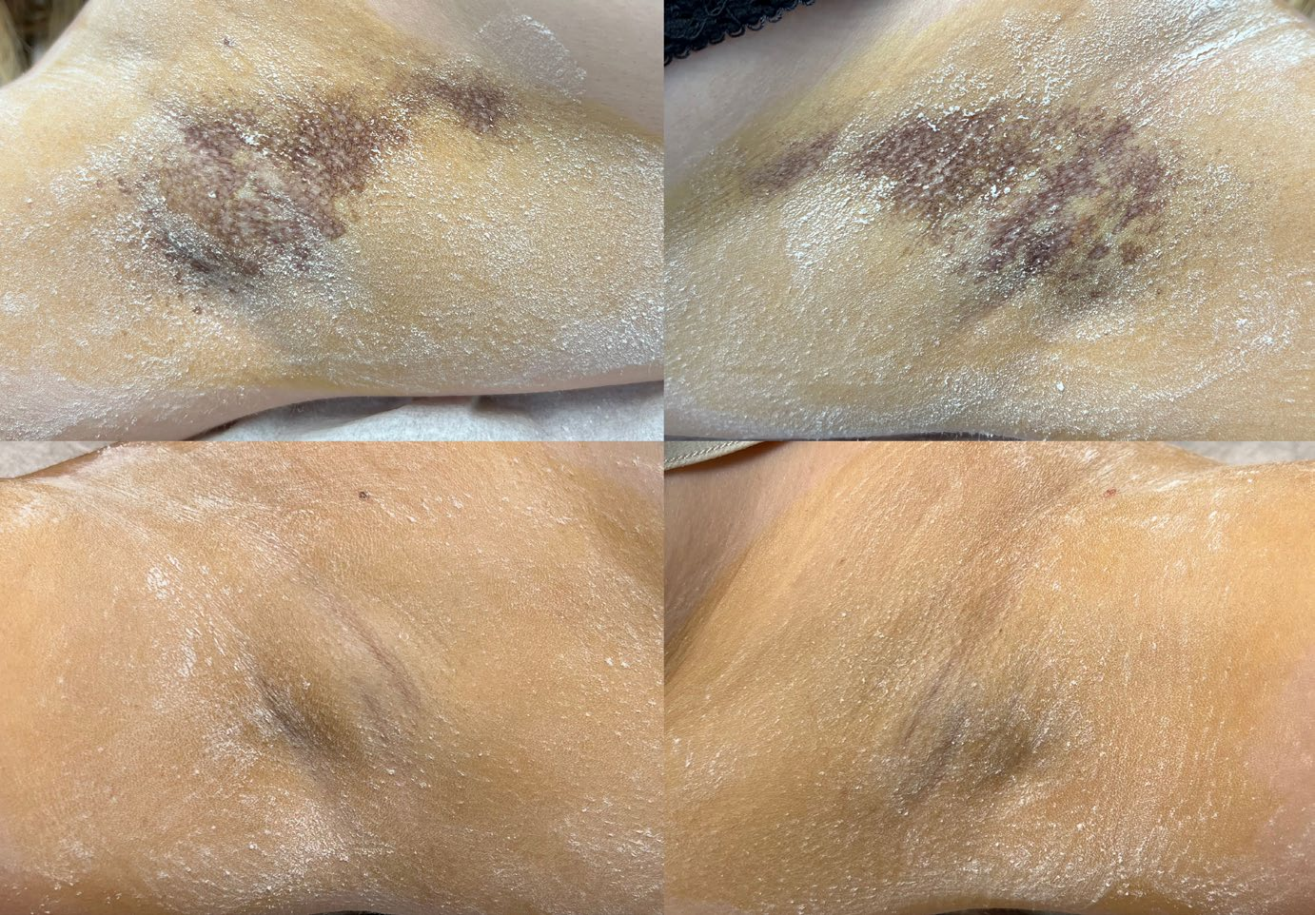
Female patient: Before (top) and after (bottom) 5 months after botulinum toxin treatment.

**FIGURE 3:**
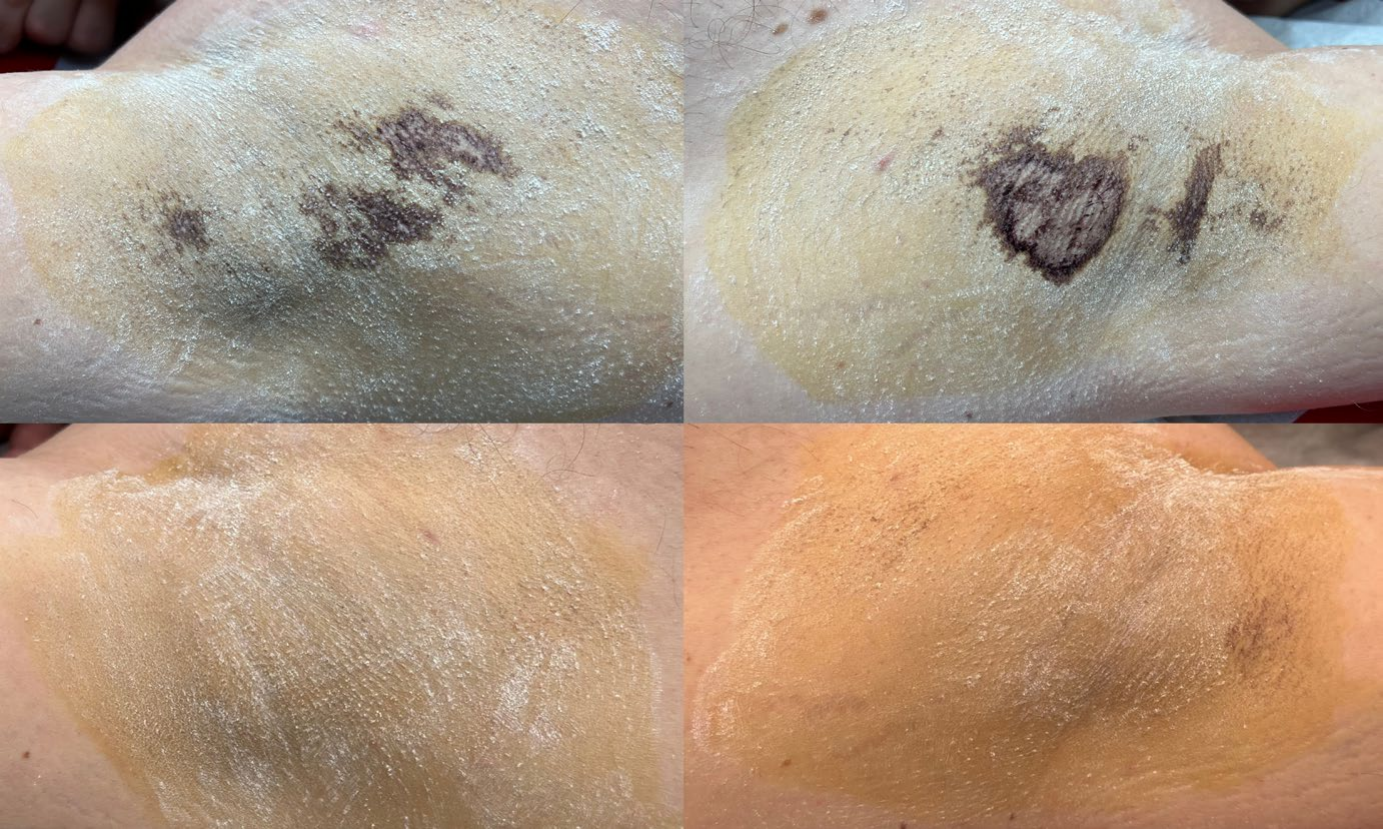
Male patient: Before (top) and after (bottom) 5 months after botulinum toxin treatment.

A 30G hypodermic needle was used, oriented at 30°–45° to the skin surface with the needle's bevel faced up. Intradermal injections at the dermal‐subcutaneous junction were delivered with injection volumes around 0.2 mL. Around 150 IE of abobotulinumtoxinA was delivered per axilla. The described protocol for botulinum toxin delivery was identical on both axillae. Following botulinum toxin delivery to both axillae, only the left axilla was then treated with a laser. A super long 1064 nm Nd:YAG pulse was delivered (PIANO mode) using a scanner (L Runner), with an irradiance setting of 1.2 W/cm^2^ and a scan area of 84 × 29 mm (SP Dynamis, Fotona, Slovenia). See Figure 1 for a diagram of the scan area. The laser procedure was performed until the surface temperature measured by the integrated noncontact thermometer (MatrixView) reached 41°C. This level of temperature was then maintained for another 3 min and then laser emission was stopped. A follow‐up visit with the iodine–starch test and photography was scheduled 1.5 months and 5 months after the treatment. See Figures 2 and 3 for the results of the iodine–starch test.

## 3 Results

The effect of botulinum toxin was consistent across both the area treated with PIANO and the area without laser treatment. The iodine–starch test revealed no differences between the two areas at both 1.5‐ and 5‐month post‐treatment.

## 4 Discussion

Historically, laser therapy was often delayed by several days or weeks after botulinum toxin had been injected. The concern was that tissue manipulation and edema from the same‐day light or energy‐based therapy could cause botulinum toxin to migrate and cause unwanted effects [27]. Additionally, there was apprehension that the skin's healing process postprocedure could accelerate the elimination of fillers or toxins [28]. It was also hypothesized that the heat generated during nonablative laser treatments could thermally or mechanically disrupt and inactivate the botulinum toxin molecule [8].

However, previous studies have demonstrated that several devices, including IPL, pulsed dye laser, diode laser, monopolar radiofrequency, and Nd:YAG laser, can be safely combined with botulinum toxin on the same day without reducing efficacy or causing unintended muscle weakening [16, 29].

In esthetic medicine, it has become standard practice to employ combined interventions targeting multiple aspects of aging skin. Administering multiple treatments in a single session not only enhances patient satisfaction but also achieves greater improvements in a shorter time frame with less overall downtime compared to multiple office visits [30]. Therefore, investigating the synergistic or potentially negative effects of combining specific treatment modalities is important.

This case series aimed to evaluate whether the 1064 nm Nd:YAG PIANO laser treatment affects the efficacy of botulinum toxin in treating axillary hyperhidrosis. Given that most manufacturers recommend keeping the toxin refrigerated, there is uncertainty whether the temperatures around 45°C could potentially affect the heat‐labile disulfide bonds and noncovalent forces between the heavy and light chains of botulinum toxin [8, 13]. Our findings show that the PIANO treatment, when applied according to the protocol (reaching an epidermal temperature of 41°C and maintaining it for 3 min), does not generate a sufficient temperature increase to impact the thermo‐labile bond. Alternatively, it suggests that this bond may be more stable than previously believed. We can theoretically deduce that the effect of laser light and the concurrent temperature increase would be even more negligible when botulinum toxin is used for wrinkle reduction, as the injection placement is deeper compared to its use in hyperhidrosis treatment.

## 5 Conclusion

This small study corroborates the findings of other research indicating that 1064 nm Nd:YAG laser treatment does not affect the efficacy of botulinum toxin. Additionally, it demonstrates that this holds true also when PIANO laser treatment is employed. Further studies with larger participant groups and various indications for botulinum toxin are necessary to further strengthen these findings.

## Conflicts of Interest

The authors declare no conflicts of interest.

## Data Availability

The data that support the findings of this study are available from the corresponding author upon reasonable request.

## References

[1] M. S. Gart and K. A. Gutowski , “Overview of Botulinum Toxins for Aesthetic Uses,” Clinics in Plastic Surgery 43, no. 3 (2016): 459–471. 27363760 10.1016/j.cps.2016.03.003

[2] M. A. S. Henning , D. Bouazzi , and G. B. E. Jemec , “Treatment of Hyperhidrosis: An Update,” American Journal of Clinical Dermatology 23, no. 5 (2022): 635–646, 10.1007/s40257-022-00707-x. 35773437

[3] L. Cooper , M. Lui , and C. Nduka , “Botulinum Toxin Treatment for Facial Palsy: A Systematic Review,” Journal of Plastic, Reconstructive and Aesthetic Surgery 70, no. 6 (2017): 833–841, 10.1016/j.bjps.2017.01.009. 28389084

[4] N. Tinastepe and B. B. Kucuk , “Botulinum Toxin for the Treatment of Bruxism,” Dermatologic Therapy 33, no. 4 (2015): 292–299. 10.1080/08869634.2015.1097296 26715152

[5] S. Aurora , “Botulinum Toxin Type A for the Treatment of Migraine,” Expert Opinion on Pharmacotherapy 7, no. 8 (2006): 1085–1095. 16722818 10.1517/14656566.7.8.1085

[6] A. W. Klein , “Complications With the Use of Botulinum Toxin,” Dermatologic Clinics 22, no. 2 (2004): 197–205. 15222580 10.1016/s0733-8635(03)00122-0

[7] L. L. Simpson , A. B. Maksymowych , J. B. Park , and R. S. Bora , “The Role of the Interchain Disulfide Bond in Governing the Pharmacological Actions of Botulinum Toxin,” Journal of Pharmacology and Experimental Therapeutics 308, no. 3 (2004): 857–864. 14617695 10.1124/jpet.103.058149

[8] S. Choudhury , M. R. Baker , S. Chatterjee , and H. Kumar , “Botulinum Toxin: An Update on Pharmacology and Newer Products in Development,” Toxins (Basel) 13, no. 1 (2021): 1–15. 10.3390/toxins13010058 PMC7828686 33466571

[9] A. Pour Mohammad , M. Gholizadeh Mesgarha , F. Seirafianpour , et al., “A Systematic Review and Meta‐Analysis of Efficacy, Safety, and Satisfaction Rates of Laser Combination Treatments vs Laser Monotherapy in Skin Rejuvenation Resurfacing,” Lasers in Medical Science 38, no. 1 (2023): 1–14, 10.1007/s10103-023-03856-5. 37776370

[10] F. Seirafianpour , A. Pour Mohammad , Y. Moradi , et al., “Systematic Review and Meta‐Analysis of Randomized Clinical Trials Comparing Efficacy, Safety, and Satisfaction Between Ablative and Non‐Ablative Lasers in Facial and Hand Rejuvenation/Resurfacing,” Lasers in Medical Science 37, no. 4 (2022): 2111–2122. 35107665 10.1007/s10103-022-03516-0

[11] D. H. Ciocon , D. Doshi , and D. J. Goldberg , “Non‐Ablative Lasers,” Current Problems in Dermatology 42 (2011): 48–55, 10.1159/000328249. 21865798

[12] J. Vartanian and S. Dayan , “Nonablative Facial Skin Tightening,” https://emedicine.medscape.com/article/844068‐overview?form=fpf.

[13] W. Huang , J. A. Foster , and A. S. Rogachefsky , “Pharmacology of Botulinum Toxin,” Journal of the American Academy of Dermatology 43, no. 2 I (2000): 249–259. 10906647 10.1067/mjd.2000.105567

[14] H. M. Seo , J. Y. Choi , J. Min , and W. S. Kim , “Carbon Dioxide Laser Combined With Botulinum Toxin A for Patients With Periorbital Syringomas,” Journal of Cosmetic and Laser Therapy 18, no. 3 (2016): 149–153. 26073121 10.3109/14764172.2015.1052517

[15] M. Zimbler and S. Undavia , “Update on the Effect of Botulinum Toxin Pretreatment on Laser Resurfacing Results,” Archives of Facial Plastic Surgery 14, no. 3 (2012): 156–158. 22801757 10.1001/archfacial.2011.1650

[16] N. L. Semchyshyn and S. L. Kilmer , “Does Laser Inactivate Botulinum Toxin?,” Dermatologic Surgery 31, no. 4 (2005): 399–404. 15871314 10.1111/j.1524-4725.2005.31105

[17] F. Al‐Niaimi , E. Glagoleva , and E. Araviiskaia , “Pulsed Dye Laser Followed by Intradermal Botulinum Toxin Type‐A in the Treatment of Rosacea‐Associated Erythema and Flushing,” Dermatologic Therapy 33, no. 6 (2020): e13976. 32633449 10.1111/dth.13976

[18] H. Pomerantz , L. Akintilo , K. Shaw , M. Lederhandler , R. Anolik , and R. G. Geronemus , “Safety Profile of Combined Same‐Day Treatment for Botulinum Toxin With Full Face Nonablative Fractionated Laser Resurfacing,” Dermatologic Surgery 47, no. 4 (2021): 500–503. 33165055 10.1097/DSS.0000000000002851

[19] E. Cuerda‐Galindo , M. A. Palomar‐Gallego , and R. Linares‐Garciávaldecasas , “Are Combined Same‐Day Treatments the Future for Photorejuvenation?,” Journal of Cosmetic and Laser Therapy 17, no. 1 (2015): 49–54. 25260050 10.3109/14764172.2014.968578

[20] N. A. Shanina , A. V. Patrushev , and A. Zorman , “Histological and Immunohistochemical Changes in Facial Skin Treated With Combined Ablative and Non‐Ablative Laser Therapy,” Journal of Cosmetic Dermatology 20, no. 11 (2021): 3509–3516. 33629512 10.1111/jocd.14023 PMC8597027

[21] L. Marini and A. Alexiou , “Photo‐Thermal Hormetic Rejuvenation With 1064 nm Nd:YAG PIANO Pulse Laser,” Journal of the Laser and Health Academy 2012 (2012): 75–79.

[22] M. Lukac , J. Kukovic , B. T. Muc , N. Lukac , and M. Milanic , “TightSculpting®: A Complete Minimally Invasive Body Contouring Solution; Part I: Sculpting With PIANO® Technology,” Journal of the Laser and Health Academy 2018, no. 1 (2018): 26–35.

[23] K. Vas , Z. Besenyi , S. Urbán , et al., “Efficacy and Safety of Long Pulse 1064 and 2940 Nm Lasers in Noninvasive Lipolysis and Skin Tightening,” Journal of Biophotonics 12, no. 9 (2019): 1–8. 10.1002/jbio.201900083 PMC7065637 31008550

[24] M. Milanic , B. T. Muc , N. Lukac , and M. Lukac , “Numerical Study of Hyper‐Thermic Laser Lipolysis With 1,064 nm Nd:YAG Laser in Human Subjects,” Lasers in Surgery and Medicine 51, no. 10 (2019): 897–909, 10.1002/lsm.23124. 31228285

[25] M. Lukac , A. Zorman , and F. Bajd , “TightSculpting®: A Complete Minimally Invasive Body Contouring Solution; Part II: Tightening With FotonaSmooth® Technology,” Journal of the Laser and Health Academy 2018, no. 1 (2018): 1–10.

[26] International Hyperhidrosis Society , “SweatHelp.org,” https://www.sweathelp.org/education‐and‐resources/starch‐iodine‐onabotulinumtoxina‐injection‐protocol‐for‐axillary‐treatment.html.

[27] N. A. Ramey and J. A. Woodward , “Mechanisms of Blepharoptosis Following Cosmetic Glabellar Chemodenervation,” Plastic and Reconstructive Surgery 126, no. 5 (2010): 248e–249e. 10.1097/PRS.0b013e3181ef822a 21042072

[28] S. B. Cho , S. J. Lee , J. M. Kang , Y. K. Kim , D. J. Ryu , and J. H. Lee , “Effective Treatment of a Injected Hyaluronic Acid‐Induced Tyndall Effect With a 1064‐nm Q‐Switched Nd:YAG Laser,” Clinical and Experimental Dermatology 34, no. 5 (2009): 637–638. 19236411 10.1111/j.1365-2230.2008.03056.x

[29] J. G. Khoury , R. Saluja , and M. P. Goldman , “The Effect of Botulinum Toxin Type A on Full‐Face Intense Pulsed Light Treatment: A Randomized, Double‐Blind, Split‐Face Study,” Dermatologic Surgery 34, no. 8 (2008): 1062–1069. 18462423 10.1111/j.1524-4725.2008.34207.x

[30] N. Langelier , K. Beleznay , and J. Woodward , “Rejuvenation of the Upper Face and Periocular Region: Combining Neuromodulator, Facial Filler, Laser, Light, and Energy‐Based Therapies for Optimal Results,” Dermatologic Surgery 42 (2016): S77–S82. 27128248 10.1097/DSS.0000000000000740

